# Patient tolerance of laryngeal electromyography: a single-center study

**DOI:** 10.25122/jml-2026-0007

**Published:** 2026-02

**Authors:** Shirley Tarabichi, Ionuţ Tănase, Mihai Alexandru Preda, Zahra Ali Chaloob, Codrut Sarafoleanu

**Affiliations:** 1 Carol Davila University of Medicine and Pharmacy, Bucharest, Romania; 2 Department of Ear, Nose and Throat (ENT) and Head and Neck Surgery, Sf. Maria Clinical Hospital, Bucharest, Romania

**Keywords:** laryngeal electromyography, tolerance questionnaire, voice disorders, procedural discomfort, EMG, electromyography, LEMG, laryngeal electromyography, TA, thyroarytenoid muscle, CT, cricothyroid muscle, VAS, visual analogue scale, BMI, body mass index, SPSS, Statistical Package for the Social Sciences, ρ, Spearman correlation coefficient

## Abstract

This observational single-center study, conducted from 2021 to 2024, evaluated patient tolerance of laryngeal electromyography (LEMG) using a standardized post-procedural questionnaire completed by 97 patients undergoing LEMG for suspected laryngeal neuromuscular dysfunction. After the procedure, patients completed a structured questionnaire designed to assess procedural tolerance across five domains: overall perception, pain intensity (visual analogue scale [VAS]), anxiety, cooperation, and post-procedural discomfort. A total tolerance score (range 0–21) was calculated for each patient. We additionally analyzed the correlation between tolerance scores and specific patient variables, including age, sex, and body mass index (BMI). Based on the total tolerance score, 32 patients (32.99%) reported excellent tolerance, 33 (34.02%) good tolerance, 9 (9.28%) poor tolerance, and 3 (3.09%) very poor tolerance. Female patients demonstrated a significantly better tolerance than male patients. No significant association was observed between tolerance score and age or BMI. No significant discomfort affecting swallowing/breathing, or voice, or any important external bleeding was reported. LEMG is generally well tolerated when performed using a standardized technique. Patient tolerance varies between individuals and appears to be influenced more by subjective factors. The structured questionnaire proved useful and provides a more extensive assessment of LEMG tolerability in clinical practice. However, because this is a single-center study, further research is needed to validate this tolerance questionnaire.

## Introduction

Electromyography (EMG) is a technique used to evaluate electrical signals generated by muscles. Motor units and muscle group recruitment are examined for abnormalities during rest and voluntary contraction [1]. Alterations in EMG patterns suggest deficiencies in neuromuscular integrity, neuromuscular function, or neuromuscular junction efficacy [2].

Laryngeal electromyography (LEMG) has been utilized for decades to assess the integrity of the laryngeal neuromuscular system by recording action potentials generated by the intrinsic laryngeal muscles during contraction [3]. The study of the electrophysiological characteristics of laryngeal function under both normal and pathological conditions began in 1944 with Weddell [4]. It was further advanced in the 1950s by researchers such as Buchthal, Faaborg-Andersen, and others [5,6]. LEMG was incorporated into the laryngological diagnosis and management of voice disorders during the late 1980s and 1990s [7,8]. Nowadays, LEMG is especially useful for differentiating between disorders affecting upper motor neurons, lower motor neurons, peripheral nerves, the neuromuscular junction, muscle fibers, as well as the laryngeal cartilages and joints [9].

LEMG is usually well tolerated by patients, does not require special preparation, and is not typically performed under local anesthesia [10]. It should not be regarded as a standalone investigation; rather, it extends the clinical examination, providing objective physiological information that supports findings from endoscopic and clinical assessments [11]. Normal EMG results suggest a mechanical problem rather than a neurological one, while abnormal waveform patterns indicate a neurological condition. Common abnormal findings include fibrillation potentials, increased insertional activity, polyphasic waves, and complex repeating discharges, and each of these findings can be associated with different causes [12,13]. Previous research has shown that LEMG findings can influence clinical management. Koufman *et al*. [14] reported an impact in 40% of patients, while Ingle *et al*. [15] found changes in management in 36% of cases.

Despite being recognized as an essential component of accurate laryngeal evaluation, the routine clinical use of LEMG remains limited. This is mainly due to persistent challenges in standardized technique, ensuring consistent interpretation, establishing diagnostic validity, and defining clear clinical applications [16].

In addition, LEMG is associated with many technical challenges arising from both clinical and anatomical factors. Variations in patient anatomy, including differences in neck anatomy, tissue density, cartilage prominence, or postoperative fibrosis, can make accurate identification of laryngeal landmarks difficult and affect needle placement. Factors related to the patient, such as discomfort, anxiety, limited cooperation, and variable tolerance for the procedure, further influence the examination. These clinical and anatomical challenges often result in signal instability and challenges in accessing target muscles [17,18]. Reported risks of EMG include bleeding, infection, vocal cord injury, and electrical sensitivity, as well as hematoma formation and airway compromise [19,20].

This study aimed to evaluate patient tolerance to laryngeal electromyography (LEMG) using a structured post-procedural questionnaire completed by a cohort of 97 patients who underwent LEMG for the assessment of laryngeal neuromuscular dysfunction. The study also analyzes the relationship between tolerance scores and patient-related variables, including age, sex, and body mass index (BMI).

## Material and Methods

A total of 109 patients who underwent LEMG for the evaluation of laryngeal neuromuscular dysfunction presented to our department between 2022 and 2024. All patients provided written informed consent to participate in the study. The study protocol was approved by the local ethics committee before patient inclusion.

A complete ENT examination was performed for each patient, including a flexible laryngoscopy. Demographic data, including age, sex, and BMI, were recorded at the time of the examination. Clinical indications for LEMG included unilateral or bilateral vocal fold movement impairment in relation to patients’ symptoms such as hoarseness, dysphagia, and difficulty breathing.

The inclusion criteria for the study were as follows:

- Patients aged ≥18 and <70 years, regardless of sex;
- Patients with dysphonia, dysphagia, chronic/acute dyspnea, surgical or non-surgical causes.
- Patients with vocal fold motion impairment, confirmed by flexible laryngoscopic evaluation;
- Patients able to understand and complete the post-procedural tolerance questionnaire;
- Patients who signed informed consent before participation.

The exclusion criteria for the study were as follows:

- Patients with acute laryngeal or cervical trauma or recent neck surgery;
- Patients with previous LEMG examination performed before the study period;
- Patients with coagulation disorders or ongoing anticoagulant treatment;
- Patients with local infection of the cervical or laryngeal region;
- Patients with insufficient clinical data or who failed to complete the tolerance questionnaire.

This observational study included 97 patients. This study was designed to evaluate patients’ tolerance to LEMG and to analyze the relationship between the tolerance score and age, sex, and BMI.

### LEMG procedure

All LEMG examinations were performed by the same examiner. Patients were examined in a sitting position, with the head flexed approximately 30 degrees. Before inserting the electrode, the anterior cutaneous region of the neck was disinfected. No local anesthetic was used in order not to interfere with the examination results. LEMG recordings were obtained using an electromyography system (Natus Viking system: EMG/EP 8-channel software, Viking EDX, 3.0.6). A 45 × 0.45 mm concentric bipolar needle electrode was used for all recordings. Each patient underwent four needle electrode insertions corresponding to bilateral examination of the thyroarytenoid (TA) (right and left) and cricothyroid (CT) muscles (right and left). A surface disk ground electrode was placed on the patient’s hand. The total duration of the LEMG investigation ranged from 25 to 45 minutes. The examination was performed bilaterally, starting on the left and proceeding to the right, at the levels of both the thyroarytenoid (TA) and cricothyroid (CT) muscles, according to standardized LEMG techniques. Anatomical landmarks were identified by palpation before each needle insertion through the cricothyroid membrane, and the correct electrode position was confirmed by acoustic and visual electromyographic feedback during phonation/deglutition.

### LEMG patient tolerance questionnaire

Patient tolerance to LEMG was evaluated using a structured post-procedural questionnaire developed specifically for this study. The questionnaire was given to all patients immediately after the examination to precisely document their subjective experience of the procedure. The average time to complete was 5 minutes. The complete patient tolerance questionnaire used in this study is provided in Supplementary file S1.

Supplementary file S1

The questionnaire comprised five distinct sections with mandatory responses, addressing both the overall perception of the procedure and specific aspects of discomfort:

1. **Overall perception of the procedure**, assessed using a six-point ordinal scale ranging from “effortless” to “unbearable,” with scores from 0 to 5.
2. **Pain intensity during the procedure** was measured using a visual analogue scale (VAS) ranging from 0 to 10, where 0 indicated no pain, and 10 indicated maximum pain. VAS values were subsequently converted to a standardized score from 0 to 5 for statistical analysis.
3. **Level of anxiety**, rated on a five-point scale from “not at all anxious” to “extremely anxious,” with scores ranging from 0 to 4.
4. **Difficulty in cooperation during the procedure**, evaluated based on the patient’s ability to follow instructions related to phonation, breathing, and swallowing, and scored from 0 to 4.
5. **Post-procedural physical discomfort**, including symptoms such as sore throat, pain at the needle insertion site, difficulty swallowing or breathing, voice changes, and significant external bleeding. This domain was scored from 0 to 3 points depending on the severity of reported symptoms.

The total tolerance score for each patient was calculated by summing the scores from all five domains, with a possible range from 0 to 21. A lower score indicated greater tolerance of the LEMG procedure, while a higher score indicated less tolerance. This score tool allowed the standardization of patient-reported tolerance measurement.

For clinical interpretation, total tolerance scores were categorized as follows:

- excellent tolerance (0–5),
- good tolerance (6–10),
- moderate tolerance (11–15),
- poor tolerance (16–18),
- very poor or intolerable tolerance (19–21).

### Statistical analysis

Descriptive analysis was performed to summarize patient characteristics and tolerance scores, with categorical variables presented as number (percentage). Statistical analysis was conducted using commercially available software (SPSS version 29.0). The total tolerance score was analyzed in relation to patient-related variables. Differences in tolerance scores between male and female patients were assessed using the Mann–Whitney U test, as the tolerance scores are ordinal and non-normally distributed. The relationships between patient age and the total tolerance score, and between BMI and the total tolerance score, were evaluated using Spearman’s rank correlation analysis. A *P* value of less than 0.05 was considered to be statistically significant, and a *P* value ≥ 0.05 indicates the absence of a statistically significant association.

## Results

Of the initial 109 patients, 97 (64 women and 33 men) who underwent LEMG in our clinic between October 2022 and December 2024 met the inclusion criteria, completed the post-procedural tolerance questionnaire, and were included in the study. The mean age was 50.1 years, and the mean BMI was 25.2 kg/m^2^. The remaining 12 patients were excluded from the study due to incomplete data or failure to meet the inclusion criteria.

The distribution of the total tolerance score (Table 1) indicated that 32 patients (32.99%) presented excellent tolerance, 33 patients (34.02%) demonstrated good tolerance, 20 patients (20.62%) showed moderate tolerance, 9 patients (9.28%) had poor tolerance, and 3 patients (3.09%) were classified as having very poor tolerance.

**Table 1:** Distribution of the total tolerance score

Tolerance	Patients	Percent (%)
Excellent	32	32.99
Good	33	34.02
Moderate	20	20.62
Poor	9	9.28
Very poor	3	3.09

The pain intensity measured by the VAS during the procedure (Table 2) exhibited an average score of 4–5. One patient (1.03%) reported minimal pain (VAS 0–1), while 11 patients (11.34%) reported mild pain (VAS 2–3). Moderate pain levels (VAS 4–5) were predominantly reported by 33 patients (34.02%). A significant number of patients reported higher pain scores, with 31 individuals (31.96%) indicating VAS values of 6–7, 11 individuals (11.34%) indicating VAS values of 8–9, and 10 individuals (10.31%) reporting severe pain (VAS 10). Elevated pain scores during the procedure were typically correlated with increased total tolerance scores.

**Table 2:** Pain level during the procedure

VAS	Score	Patients	Percent (%)
0-1	0	1	1.03
2-3	1	11	11.34
4-5	2	33	34.02
6-7	3	31	31.96
8- 9	4	11	11.34
10	5	10	10.31

Regarding anxiety (Table 3), 12 patients (12.37%) indicated the absence of anxiety, while another 12 patients (12.37%) reported mild anxiety during the procedure. 26 patients (26.80%) reported moderate anxiety, while high levels of anxiety were common, with 20 patients (20.62%) identifying as very anxious and 27 patients (27.84%) as extremely anxious during the examination.

**Table 3:** Distribution of patient anxiety levels during LEMG

Anxiety	Score	Patients	Percent (%)
Not at all anxious	0	12	12.37
Mildly anxious	1	12	12.37
Moderately anxious	2	26	26.80
Very anxious	3	20	20.62
Extremely anxious	4	27	27.84

Difficulties with cooperation (Table 4) were reported as absent in 41 patients (42.27%), while 29 patients (29.90%) reported mild difficulty. Moderate difficulty during the procedure was noted in 15 patients (15.46%), and significant difficulty was observed in 9 patients (9.28%). In 3 patients (3.09%), cooperation was not possible; however, the examination was still successfully completed.

**Table 4:** Cooperation difficulties during the procedure

Cooperation difficulties	Score	Patients	Percent (%)
No difficulty	0	41	42.27
Mild difficulty	1	29	29.90
Moderate difficulty	2	15	15.46
Significant difficulty	3	9	9.28
Unable to cooperate	4	3	3.09

Regarding the physical discomfort after the procedure (Table 5), most patients (89.69%) experienced either no (45 patients) or mild (42 patients) post-procedural discomfort, while 10 patients (10.31%) reported moderate discomfort. No significant discomfort affecting breathing, swallowing, or voice, or any important external bleeding associated with the LEMG procedure, was reported.

**Table 5:** Physical discomfort after the procedure

Physical discomfort	Score	Patients	Percent (%)
No discomfort	0	45	46.39
Mild discomfort (mild sore throat or mild pain at the puncture site)	1	42	43.30
Moderate discomfort (moderate sore throat or moderate pain without functional limitations at the puncture site)	2	10	10.31
Significant discomfort affecting swallowing/voice or important external bleeding	3	0	0

Regarding the general perception of the LEMG procedure (Table 6), 26 patients (26.80%) classified the examination as very easy, while 31 patients (31.96%) evaluated it as easy. A moderately difficult experience was reported by 27 patients (27.84%). 10 patients (10.31%) found the procedure difficult, while 3 patients (3.09%) found it very difficult. No patient deemed the procedure intolerable.

**Table 6:** General perception of the LEMG procedure

General perception	Score	Patients	Percent (%)
Very easy	0	26	26.80
Easy	1	31	31.96
Moderately difficult	2	27	27.84
Difficult	3	10	10.31
Very Difficult	4	3	3.09
Unbearable/Insupportable	5	0	0

### Relationship between tolerance score and patient variables

#### Gender

Differences in total tolerance scores between female and male patients were analyzed using the Mann–Whitney U test. A *P* value < 0.05 was considered statistically significant. Female patients showed significantly better tolerance to the LEMG procedure as compared to male patients, as indicated by lower total tolerance scores (*P* = 0.01) (Table 7).

**Table 7:** Correlation between sex and total tolerance score

Gender	Patients	Median tolerance score	*P* value
Female	64	1.0 (0-2)	0.01
Male	33	1.0 (1-3)	

#### Age

The relationship between age and total tolerance score was assessed using Spearman correlation. No statistically significant correlation was observed between patient age and total tolerance score (Spearman’s ρ = −0.09, *P* = 0.37; Table 8). This finding indicates that age was not a meaningful determinant of patient tolerance in the studied cohort (*P* value ≥ 0.05).

**Table 8:** Correlation between age and total tolerance score

Variable	Patients	Correlation coefficient (ρ)	*P* value
Age (years)	97	−0.09	0.37

#### BMI

The mean BMI of the study population was 25.2 kg/m^2^. Analysis of the relationship between BMI and the total tolerance score (Table 9) using Spearman rank correlation (ρ = 0.12, *P* = 0.24) demonstrated no statistically significant association between BMI and patient-reported tolerance to LEMG (*P* ≥ 0.05). These findings indicate that BMI did not affect subjective tolerance to the procedure in the present cohort.

**Table 9:** Correlation between BMI and total tolerance score

Variable	Patients	Mean BMI (kg/m^2^)	Correlation coefficient (ρ)	*P* value
BMI	97	25.2	ρ = 0.12	0.24

## Discussion

LEMG is an investigation that records electrical signals from the laryngeal muscles during voluntary and involuntary contraction to evaluate neurological impairment. It provides supplementary information on laryngeal function to validate or challenge the diagnosis obtained during clinical examination and laryngeal fibroscopy, and it is also a useful tool for evaluating the possibility of nerve recovery [9].

The present study provides a detailed assessment of patient tolerance to LEMG using a structured, procedure-specific questionnaire applied to a cohort of 97 patients. The questionnaire evaluates the pain felt during the investigation, anxiety, cooperation, and post-procedural discomfort. Our findings indicate that LEMG is generally well tolerated: 65 patients (67.01%) of 97 reported excellent or good tolerance, 20 (20.62%) moderate, and 12 (12.37%) poor or very poor. No patient classified the procedure as insupportable. This makes LEMG still a valuable diagnostic tool for evaluating laryngeal neuromuscular dysfunction.

Studies in the literature have established that LEMG is an important adjunct in the evaluation of vocal cord palsy, particularly for differentiating neurogenic from mechanical causes and providing prognostic information regarding neural recovery [21,22]. Although it is an investigative tool with enormous potential, it is often perceived as inconvenient or technically demanding. This perception has led to its limited use and lack of acceptance in standard clinical practice. Other published studies characterize patient tolerance in general terms [23]. The questionnaire used in this study provides a more thorough and standardized evaluation of patient tolerance, including significant variables such as patient anxiety and cooperation, which are particularly important.

Pain perception is an important component of tolerance. Pain and tolerance during medical procedures are complex, highly individual experiences that depend on factors such as physical sensation, psychological response, and patient expectations. Even when a surgery is performed using a standardized approach under identical conditions, patients may exhibit substantially different levels of discomfort and tolerance. Anticipatory anxiety, previous medical experiences, personal pain tolerance, and the perception of procedural presence all impact the experience and reporting of discomfort. So, pain intensity alone does not accurately reflect procedural tolerance, which additionally includes the patient’s capacity to manage and collaborate during the test [24–26].

Pain intensity during LEMG investigation was assessed using a VAS. One patient reported minimal pain (VAS 0–1) during the procedure. Mild (VAS 2-3) to moderate (VAS 4-5) pain levels were reported by 44 patients (45,36%), while higher pain scores (VAS ≥ 6) were reported by 52 patients; no patient classified the examination as intolerable.

This study also analyzed the total tolerance score according to gender. Although the median tolerance score was similar between genders, the distributions of scores differed significantly, indicating that male patients experienced greater discomfort during the investigation than female patients. It was observed that female patients demonstrated better tolerance to the LEMG procedure, while male patients more frequently reported higher (worse) tolerance scores. Specifically, 65 patients (67.01% of the total cohort) reported excellent or good tolerance, a proportion higher among female patients than among males. In contrast, poor or very poor tolerance was observed in 12 patients (13.37%), and it was more commonly observed in male patients. Previous research had demonstrated sex-related differences in pain perception and coping mechanisms [27,28], and our findings indicate that these differences may contribute to variations in patient tolerance to LEMG.

In contrast, patient age was not correlated with variations in tolerance scores. This suggests that LEMG can be performed for any age without a significant impact on patient tolerance. This data is particularly relevant given that vocal fold paralysis and other laryngeal neuromuscular disorders commonly impact the elderly population. Specialized studies also indicate that other individual characteristics, rather than age, may affect procedural tolerance [23].

In this study, patients reported a high percentage of anxiety. 47 patients (48.46 %) reported very high or extreme anxiety, while 38 of them (39.17 %) reported experiencing mild to moderate anxiety during the procedure. Only 12 patients (12.37%) indicated the absence of anxiety. The relatively high levels of anxiety reported by a substantial proportion of patients may be explained by the nature of the procedure itself, given that it is an invasive investigation that is performed without any anesthesia. The fact that the patient knows that the needle will be inserted in an extremely sensitive anatomical region makes him already anticipate the discomfort, and increases the level of anxiety. In addition, the awareness of the need for active cooperation during the investigation can also contribute to increasing the level of anxiety. These factors may influence the patient’s perception of the procedure, even in the absence of severe pain, and may partially explain the levels of anxiety observed in this study. This observation corresponds to findings from other invasive investigations, where anticipatory anxiety has been shown to affect pain perception and general tolerance [24].

As previously noted, during LEMG, active patient participation is required for phonation, breathing, and swallowing; thus, anxiety can negatively affect cooperation and signal quality. In our study, regarding patient cooperation during the procedure, it was generally good. 70 patients (72.17%) reported feeling no difficulty or only slight difficulty in cooperating. An additional 15 patients (15.46%) reported moderate difficulty, while 9 patients (9.28%) experienced significant difficulty cooperating during the examination. In 3 patients (3.09%), cooperation was not possible, but the examination was completed. These findings highlight the importance of comprehensive pre-procedural counseling and clear communication to reduce patient anxiety, facilitating cooperation during the investigation, and improving the procedural experience.

Although some patients reported higher levels of pain or anxiety during the procedure, post-procedural symptoms were generally mild and transient. In the present study, 45 patients (46.39 %) reported no post-procedural discomfort, while 42 (43.30 %) reported mild sore throat or mild pain at the needle insertion site. Only 10 patients (10.31 %) had moderate discomfort after the investigation. There were no major complications (significant bleeding from the puncture site, dysphonia, dysphagia) that required medical intervention or hospitalization.

Crespo [17] reported that after addressing the TA muscles, one patient presented with a laryngeal hematoma that partially occluded the laryngeal lumen. In another study involving 89 patients, Lu *et al*. [29] examined hemodynamic stability during LEMG procedures and found that mild but significant increases in diastolic blood pressure and pulse were associated with occasional transient hemodynamic reactions, such as near syncope, in a small proportion of patients. However, none of the patients in our study developed complications after completion of the investigation.

These findings emphasize that, despite the invasive nature of LEMG and the discomfort experienced by some patients during the procedure, the investigation is safe, generally well tolerated, and not associated with major postprocedural complications. This should be clearly communicated to patients to improve confidence, cooperation, and the overall experience of the procedure.

The relationship between BMI and patient tolerance to LEMG did not show a statistically significant association with total tolerance score. Patients with higher BMI did not report worse tolerance than those with lower BMI, indicating that BMI was not a determinant of patient-reported tolerance in this cohort. In contrast, BMI can influence the technical aspects of LEMG performance. Increased soft tissue thickness in patients with a higher BMI may make it more difficult to palpate anatomical landmarks and position the needle, potentially complicating the procedure or increasing the technical difficulty [30].

O’Connell *et al*. [23] evaluated the pain associated with LEMG needle insertion using standardized tools, including the VAS and the McGill Pain Questionnaire. In their study, overall patient-reported pain levels were predominantly mild to moderate, supporting the view that although LEMG is performed while the patient is awake and without local anesthesia, it is not usually associated with severe or intolerable pain.

Several strengths of this study should be highlighted. All LEMG examinations were performed by a single experienced examiner, using a standardized protocol for each patient. This approach minimized variability and allowed for a more reliable assessment of patient tolerance. In a recent study, pain and tolerance related to LEMG were reported based on examinations performed by different clinicians across several centers, which may introduce heterogeneity in technique and patient experience [31]. The standardized methodology used in the present study, therefore, represents an important advantage. In addition, the development of this tolerance questionnaire addresses an important gap in the literature.

The limitations of this study should also be emphasized. The study was conducted at a single center, which may limit generalizability. Furthermore, although the questionnaire was systematically designed, it requires future validation in larger, independent cohorts. Psychological factors such as pre-procedural anxiety or previous medical experiences were not formally assessed and may have influenced patient-reported tolerance.

## Conclusion

These results demonstrate that LEMG is a generally well-tolerated diagnostic procedure, given that the majority of patients reported excellent or good tolerance, and the severe discomfort was uncommon, and no major complications were observed.

Gender was identified as a significant factor influencing tolerability, with female patients demonstrating better overall tolerability than male patients, whereas patient age was not associated with tolerability. Even when the investigation was performed using the same standardized technique and under identical conditions, individual pain perception varied considerably. This variability underscores the influence of patient-specific factors, such as pain sensitivity and psychological responses, on the procedure’s overall tolerability. These findings suggest that a detailed, step-by-step explanation of the investigation and of the sensations expected during the procedure could reduce anxiety, improve the patient’s perception of the examination, and increase overall tolerance of LEMG. However, given the single-center design and the sample size, further studies involving larger patient populations and multicenter cohorts are necessary to confirm these findings, validate the tolerance questionnaire, and improve the generalizability of the results. Future research should also explore additional clinical and psychological factors that may influence patient tolerance and identify strategies to further optimize comfort during LEMG.
