# Supplementary material for: Patient tolerance of laryngeal electromyography: a single-center study
Source: J Med Life. 2026 Feb;19(2):148–54. doi: 10.25122/jml-2026-0007 (PMC13059430; doi:10.25122/jml-2026-0007)
Supplement: Supplementary file 1 [file JMedLife-19-148-s001.pdf]

Name..

LEMG Patient Tolerance Questionnaire

▲ **Higher score = worse tolerance** | ▼ **Lower score = better tolerance**

1 Overall Perception of the Procedure *0 – 5 points*

PATIENT ANSWER	SCORE
Very easy	**0**
Easy	**1**
Moderately difficult	**2**
Difficult	**3**
Very difficult	**4**
Unbearable / Insupportable	**5**

2 Pain Level During the Procedure (0–10 scale) *0 – 5 points*

Rate your pain: 0 = no pain at all → 10 = worst imaginable pain

0	1	2	3	4	5	6	7	8	9	10

VAS PAIN (0–10)	SCORE
0 – 1	**0**
2 – 3	**1**
4 – 5	**2**
6 – 7	**3**
8 – 9	**4**
10	**5**

3 Anxiety or Discomfort *0 – 4 points*

PATIENT ANSWER	SCORE
Not at all anxious	**0**
Mildly anxious	**1**

© 2026 by the authors. This article is an open access article distributed under the terms and conditions of the Creative Commons Attribution (CC BY 4.0) license.

Moderately anxious	2
Very anxious	3
Extremely anxious	4

4 Cooperation Difficulty *0 – 4 points*

PATIENT ANSWER	SCORE
No difficulty	0
Mild difficulty	1
Moderate difficulty	2
Significant difficulty	3
Unable to cooperate	4

5 Physical Discomfort After the Procedure *0 – 3 points*

PATIENT ANSWER	SCORE
No discomfort	0
Mild throat soreness OR mild pain at puncture site	1
Moderate discomfort (2 or more symptoms)	2
Significant discomfort affecting swallowing / voice / bleeding	3

TOTAL SCORE = Q1 + Q2 + Q3 + Q4 + Q5

Minimum: **0** | Maximum: **21**

INTERPRETATION OF TOTAL SCORE

TOTAL SCORE	INTERPRETATION
0 – 5	Excellent tolerance
6 – 10	Good tolerance
11 – 15	Moderate tolerance
16 – 18	Poor tolerance
19 – 21	Very poor / intolerable tolerance

© 2026 by the authors. This article is an open access article distributed under the terms and conditions of the Creative Commons Attribution (CC BY 4.0) license.
